# Behavioural and Electrophysiological Response of *Anastrepha fraterculus* (Diptera: Tephritidae) to a γ-Lactone Synthetic Semiochemical

**DOI:** 10.3390/insects14020206

**Published:** 2023-02-18

**Authors:** Lucía Goane, Beatriz N. Carrizo, María Josefina Ruiz, Guillermo E. Bachmann, Fabian H. Milla, Diego F. Segura, Dan Kuzmich, Spencer Walse, María Teresa Vera

**Affiliations:** 1 Facultad de Agronomía, Zootecnia y Veterinaria, Universidad Nacional de Tucumán, San Miguel de Tucumán T4000, Argentina; 2 Consejo Nacional de Investigaciones Científicas y Técnicas (CONICET), Ciudad Autónoma de Buenos Aires C1425FQB, Argentina; 3 INTA Estación Experimental Agrícola Famaillá, Famaillá T4132, Argentina; 4 Instituto de Genética “EA Favret”, INTA, GV-IABIMO, CONICET, Partido de Hurlingham B1686, Argentina; 5 San Joaquin Valley Agricultural Sciences Center, Agricultural Research Service, United States Department of Agriculture, Parlier, CA 93648, USA

**Keywords:** (±)-epianastrephin, (±)-anastrephin, (±)-*trans*-tetrahydroactinidiolide, dimethyl, physiological status, field cage experiments, IPM, species-specific attractants

## Abstract

**Simple Summary:**

Specific attractants are one of the best allies in insect-integrated pest management. At present, monitoring of *Anastrepha fraterculus*, a species of great economic importance in South America, relies on food-based attractants with poor selectivity and high cost, from an operational perspective. Here, we analyzed the response of *A. fraterculus* to different semiochemicals recently synthesized. This response was conditioned by the physiological condition and sex of the flies. To our knowledge, this is the first report that clearly shows a sexual role of epianastrephin in *A. fraterculus*. A structurally related, naturally occurring γ-lactone, (±)-trans-tetrahydroactinidiolide, gave a promising performance since flies responded to it as they did to epianastrephin; however, it has an advantage over epianastrephin since it is easier to synthesize and could serve as an effective early-detection attractant, particularly in pest-free areas such as entry ports in importing countries. Variations of attraction according to flies’ physiological status and age are also an important finding for pest management since they contribute to improving the available attractants and trapping effectiveness of *A. fraterculus*. We suggest that open field evaluations in commercial orchards will ultimately determine the possibilities of implementing these semiochemicals as new attractants for *A. fraterculus*.

**Abstract:**

Attractants are a powerful tool for pest management. The lack of specific attractants for the South American fruit fly, *Anastrepha fraterculus*, a complex of cryptic species of great economic importance in South America, makes it difficult to monitor the pest in the field. The γ-lactone male sex and aggregation pheromones of several *Anastrepha* species, naturally released in a 7:3 epianastrephin to anastrephin ratio, and a structurally related naturally occurring γ-lactone ((±)-trans-tetrahydroactinidiolide) with gem-dimethyl groups (dimethyl) at C(4), were evaluated as potential attractants of this species. Different age and mating conditions of *A. fraterculus* males and females were evaluated during electroantennography (EAG) and field cage experiments in which polymeric lures were deployed to contain 100 mg of attractant. Epianastrephin and dimethyl were EAG+ for all fly conditions, with epianastrephin eliciting the highest response for both sexes and immature flies showing greater responsiveness than mature flies. In the field cage experiments, immature flies were only attracted to leks; virgin females were attracted to leks, dimethyl, and both epianastrephin-anastrephin formulations (95 and 70 wt.% epianastrephin); mature-mated males were attracted to leks, dimethyl and 70 wt.% epianastrephin; and mature-mated females were only attracted to leks. Our bioassays showed a promising performance of the analog dimethyl since it elicited the same response as epianastrephin, requires fewer steps to synthesize, and contains one less chiral center than the natural pheromones. The attraction to leks was recorded for all mating conditions and ages of flies and suggests that air-borne volatiles of calling males contain cues that could act as sensory traps. The addition of any of these compounds in the synthetic attractants may result in a greater attraction and thus deserves further evaluation. Dose-response experiments will provide additional information to move a step forward and validate the results obtained in open-field conditions.

## 1. Introduction

Insects exploit chemical information to feed, mate, find hosts, evade enemies, and execute a myriad of other behavioral strategies. Responses to these chemical signals vary according to environmental factors, previous experience, physiological status, or combinations of all of these [1]. Attraction to chemical stimuli has been the subject of study for its application in pest monitoring and control [2,3,4,5,6,7,8,9]. Pheromone attractants, species-specific by definition, may function with sexual or physiologic specificity to aid detection, population monitoring, and eradication programs [10]. The current availability of efficient methods to synthesize semiochemicals allows for improving their implementation and contributes to elucidating the evolutionary and ecological aspects of the target insect.

Among Tephritidae, sexual attractants with high specificity (male lures) as trimedlure, methyl-eugenol, and cue-lure are used as attractants in official eradication programs for early detection of exotic species, such as *Ceratitis capitata*, *Bactrocera dorsalis B. tryoni,* and *Zeugodacus cucurbitae* [7,11,12,13,14]. Given the relevance of semiochemicals as fly attractants, research to analyze their specificity and biological effectivity, mainly for the *Anastrepha* group, is required [9]. Recently, Walse and Kuzmich [15] reported the racemic synthesis of γ-lactone semiochemicals, including male sex and aggregation pheromones of several *Anastrepha* species (i.e., (±)-epianastrephin and (±)-anastrephin). Furthermore, a structurally related naturally occurring analog where the chiral center at C(4) of epianastrephin and anastrephin is substituted with gem-dimethyl groups, (±)-*trans*-tetrahydroactinidiolide [16,17], was also synthesized [15]. These reports opened the possibility of developing specific attractants for members of this genus.

Sexual attractants have been relatively unexplored for the South American fruit fly, *A. fraterculus* [18]. The current monitoring of this species is usually based on food baits [19]. *Anastrepha fraterculus* is a complex of cryptic species [20,21] of great economic importance for fruit production and export from South America [22,23,24]. The species complex is composed of at least eight different morphotypes [25,26] with a wide distribution and a host range that includes many plant species of economic importance [27,28].

As in other *Anastrepha* species, *A. fraterculus* males congregate in courtship areas (leks) where they release pheromones to attract females [22,29]. The chemical composition of the male air-borne volatiles varies according to the diet, breeding conditions of males, and morphotypes [18,30,31,32,33,34], the main components of which are (E)-β-ocimene, n-nonanal, (Z)-3-nonen-1-ol, benzoic acid, suspensolide, (Z,E)-α-farnesene, (E,E)-α-farnesene, limonene, anastrephin, and epianastrephin. Six of these compounds triggered an antenna response in virgin females [18]. However, antennae response does not always imply a behavioral response of the flies, and some studies suggest that the antennae’s ability to perceive odors can vary according to the age and mating status of the flies [35]. Detecting the responses of *A. fraterculus* at different physiological conditions to such compounds will contribute to managing its populations. Additionally, this topic gains relevance in the context of the incipient speciation processes affecting the species of this group.

The present study was conducted to determine the attractiveness of *A. fraterculus* males and females of different physiological conditions to three γ-lactone semiochemicals (anastrephin, epianastrephin, and the synthetic γ-lactone analog dimethyl) through electroantennography evaluation and behavioral tests carried out under field cage conditions.

## 2. Materials and Methods

### 2.1. Individuals

Flies used to evaluate attractiveness to synthetic semiochemicals were obtained from a colony of the Brazilian 1 morphotype within the *A. fraterculus* cryptic species complex kept at IGEAF (INTA, Buenos Aires, Argentina). This colony was initiated in 1997 with pupae obtained from infested guavas collected in Tafí Viejo (Tucumán, Argentina). Larvae were fed a diet with equal parts of sugar, brewer’s yeast, and wheat germ. Upon emergence, a group of flies was sorted by sex in order to allow them to complete their sexual maturation without the possibility to mate while, for another group of flies, males and females were kept together and allowed to mate. Adults were fed a diet based on sugar and yeast extract (Bionis^®^, Biorigin, São Paulo, Brazil) (3:1 ratio) and provided water *ad libitum* before evaluation.

For electroantennography studies (EAG), sexually immature unmated flies (2–5 days old), sexually mature unmated flies (10–14 days old), and sexually mature mated flies (10–14 days old) of both sexes (females and males) were included, totaling six treatments (i.e., fly conditions). For field cage experiments, sexually immature flies (2–3 days old) of both sexes, sexually mature unmated females (10–14 days old), and sexually mature mated males and females were evaluated, totaling five treatments (i.e., fly conditions). To simplify reading, we will refer to sexually immature unmated flies as immature flies, to sexually mature unmated flies as virgin flies, and to sexually mature mated flies as mated flies.

### 2.2. Synthetic Semiochemicals

The molecules (±)-epianastrephin and (±)-anastrephin were synthesized according to recent methods [15] and formulated together in a slow-release polymeric matrix (vinyl chloride) device [36] with different proportions of both compounds (see below). According to the methods described above, a naturally occurring pheromone analog, where the chiral center at C(4) is substituted with gem-dimethyl groups [15], (±)-*trans*-tetrahydroactinidiolide (dimethyl, henceforth), was prepared and formulated with the advantage of having one less chiral center and requiring less synthetic operations than the natural pheromones. For EAG evaluation, epianastrephin was evaluated purely to confirm whether it elicited an antenna response and to compare the antennae depolarization of *A. fraterculus* exposed to dimethyl (EAG unknown). Anastrephin was not considered in EAG experiments because, even though it is detected in the pheromone of *A. fraterculus* males, it did not elicit consistent antennae depolarization in previous studies [18]. For field cage experiments, epianastrephin and anastrephin were formulated in two ratios (70:30 and 95:5; referred to hereafter as epianastrephin 70 and epianastrephin 95) and dimethyl was tested alone. In all cases, the semiochemicals were embedded in a slow-release polymeric matrix at a dose of 100 mg as detailed in [36].

### 2.3. Experimental Procedures

#### 2.3.1. Electroantennography

Electroantennographic records (EAG) were performed to evaluate the detectability of dimethyl and epianastrephin by different sexes and the physiological conditions of *A. fraterculus*. Semiochemicals were embedded in a polymeric matrix. A piece of 0.01 g of this matrix was placed in a glass Pasteur pipette. This preparation was used as a stimulus, while a second, empty Pasteur pipette was used as a negative control. The sensitivity of the antennae was tested using a signal amplifier (IDAC2, Syntech, Hilversum, The Netherlands) and data acquisition software (GC-EAD 2014 v. 1.2.5, Syntech, Ockenfels, Germany). A fly head was excised and mounted through its base on the tip of an elongated glass capillary filled with phosphate buffer saline solution (PBS) 1X, which contained the indifferent electrode. The tip of one antenna contacted the recording electrode. The antenna received a constant flow of filtered and humidified air at 16.0 mL/min. Glass and Tygon tubing were used for all air movement. The tip of the Pasteur pipette carrying the polymeric matrix ended in the mixing tube carrying the stimulus to the antenna in the air stream. The base of this pipette was connected to a flexible tube that was connected to a solenoid valve. The same system was used for the control but without the polymeric matrix. For each antenna, a series of air pulses (puffs) was delivered as follows: air, dimethyl, epianastrephin, air, epianastrephin, dimethyl, air. The dimethyl and epianastrephin puffs were alternated with each new antenna. Each puff was applied for 1.0 s, and the time between consecutive puffs was 60 s in order to minimize potential sensorial adaptation of the antenna. The depolarization of antennae was estimated through the amplitude of the electroantennographic peaks, and the two values recorded for each stimulus were averaged within the same series. Thus, three values were obtained, control, dimethyl, and epianastrephin, for each antenna. Each antenna was considered a replicate, with ten replicates performed per fly condition.

#### 2.3.2. Field Cage Experiments

The attractiveness behavior of *A. fraterculus* to the semiochemicals was studied under semi-natural conditions. The study was carried out at the experimental field of Facultad de Agronomía, Zootecnia y Veterinaria, Universidad Nacional de Tucumán, Argentina, where five field cages 30 m apart from each other were positioned in a shaded area. Field cages consisted of cylindrical cages (3 m in diameter and 2 m high) made of synthetic mesh walls hung from a metal structure. Lemon trees (*Citrus limon*) of approximately 1.8 m in height and 1.5 m in diameter were placed in the center of each cage to provide resting surfaces for the flies. Experiments were carried out from October 2020 to April 2021 and from October 2021 to March 2022 on days with optimal weather conditions for the flies. The average temperature ranged from a minimum of 17 °C to a maximum of 30 °C. In addition to semiochemicals, a natural pheromone release unit (lek) consisting of 10 confined, sexually mature, live males (12–15 days old) that naturally released pheromone was also included. The lek contained cotton soaked in water to avoid dehydration of males and was placed in a control cage.

Three independent experiments were performed considering each one of the fly conditions: immature flies (males and females), virgin females, and mated flies (males and females). For each experiment, two McPhail traps, one with the stimuli (one of the evaluated synthetic semiochemicals or the lek) and one without it (control) were hung 15 cm from the roof of the cage. McPhail traps had in their base 250 mL of water with a thin layer of mineral oil. Water-soaked cotton was placed on the trees to prevent flies from responding to the traps in search of water. The stimuli hung from the top of the trap. One hundred flies of a given sex, age, and mating status were released inside the cage at sunset from 6:30 pm to 7:00 pm to ensure fly acclimatization before the time of sexual activity (early morning for *A. fraterculus* Brazilian 1 morphotype). The following day, about 18 h later, between 12:00 am and 2:00 pm, traps were removed from the cage. The number of flies caught per trap was recorded. Flies that were not caught in the traps were removed from the cages, counted, and discarded. The same day, at sunset and after cage-cleaning, another group of flies was released. Traps were rotated among cages to ensure that all stimuli were evaluated in all cages. In the first experiment of immature flies (males and females), in addition to the synthetic semiochemicals and the lek, a positive control cage with a food-based attractant torula yeast (Susbin^®^, Mendoza, Argentina), was set up. We hypothesized that immature young flies would be attracted to a food source rather than a sexual stimulus. Therefore, this cage allowed us to ensure that flies were active (positive control) in the case of no response to the other stimuli. For the second and third experiments, we did not expect this to occur, therefore the cage with a food-based attractant was not included.

### 2.4. Data Analysis

#### 2.4.1. Electroantennography

To analyze the response (amplified signals expressed as mV) of *A. fraterculus* to the semiochemicals, control values were subtracted from the average values obtained for dimethyl and epianastrephin, respectively. This procedure allowed us to dismiss the effects of air and background noise. A general linear model was built with these corrected values as the response variable. Sex, semiochemical, physiological condition of flies (age and mated status), and their interactions (excluding sex x physiological condition and sex x semiochemical x physiological condition) were included in the model. Normality (Q-Q plot) and homogeneity of standardized residuals (residuals plot) were verified [37]. Multiple comparisons were performed with Di Rienzo, Guzmán, and Casanoves (DGC) test [38]. Analyses were conducted using R interface of Infostat [39].

#### 2.4.2. Field Cage Experiments

The Wilcoxon test for paired samples was used to compare the number of flies caught by the trap containing the semiochemical vs. the control trap (without the attractant). Furthermore, a generalized lineal model with negative binomial distribution was built to compare the attractiveness among stimuli (synthetic semiochemicals and leks). Fly sex, semiochemical, and their interactions were included as fixed factors, and the proportion of *A. fraterculus* caught by traps containing the sexual attractant (number of flies caught by the trap containing the attractant/control trap) as the response variable. Model fit was evaluated by the ratio of the deviance and its degree of freedom. Analyses were separately performed for each fly’s physiological condition with Infostat [39].

## 3. Results

### 3.1. Electroantennography

**Figure 1:**
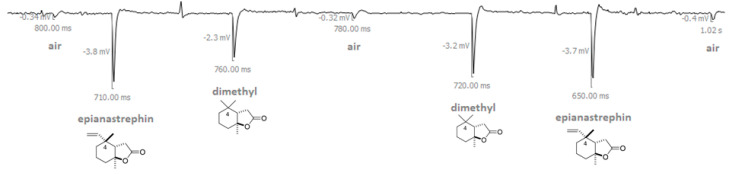
Example of electroantennographic output when an antenna of a sexually immature male of *Anastrepha fraterculus* was exposed to a series of air pulses (puffs) in this order: air, epianastrephin, (±)-*trans*-tetrahydroactinidiolide (dimethyl), air, (±)-*trans*-tetrahydroactinidiolide (dimethyl), epianastrephin, air (see Material and Methods section for more details). The amplitude of depolarization is displayed in millivolts (mV), and the time elapsed between the onset and the maximum of depolarization is displayed in milliseconds (ms).

**Figure 2:**
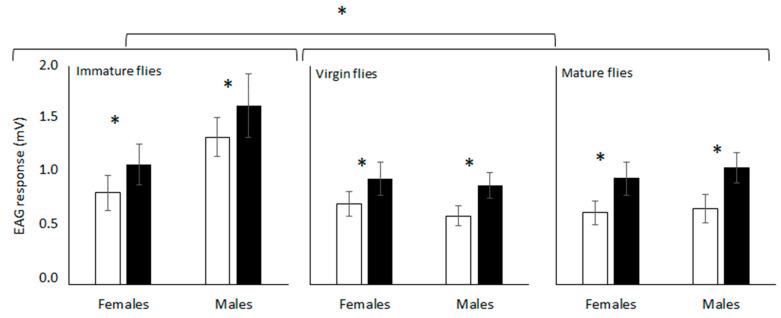
Average response (amplified signals expressed as mV) of *Anastrepha fraterculus* antennae from different physiological fly conditions to (±)-*trans*-tetrahydroactinidiolide (dimethyl; white bars) and epianastrephin (black bars) in electroantennography. Asterisk denote significant differences (DGC, α 0.05).

Both dimethyl and the synthetic epianastrephin were found to be EAG active for all the flies’ conditions evaluated (Figure 1). The antennal response differed significantly among semiochemicals and physiological conditions of the flies but did not differ between sexes (Table 1). No significant interaction was found among the factors (Table 1). Epianastrephin elicited the highest EAG response independently of the sex, and this response was higher for immature females and males (Figure 2).

### 3.2. Field Cage Experiments

#### 3.2.1. Immature Females and Males

**Figure 3:**
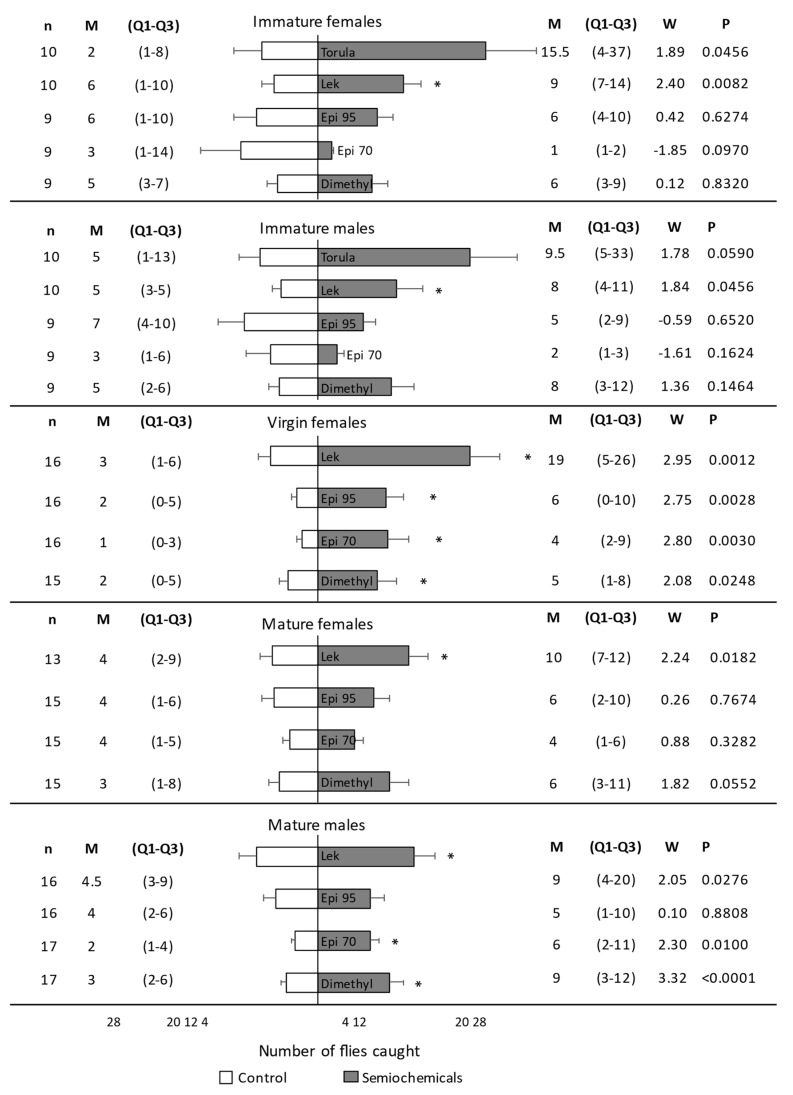
Number of *Anastrepha fraterculus* caught in traps with or without the stimulus under field cage experiments. Immature flies, mature-virgin females, and mature-mated flies were considered in separate experiments. *Trans*-tetrahydroactinidiolide (Dimethyl) and two epianastrephin-anastrephin formulations were considered: 95 wt.% epianastrephin (Epi 95) and 70 wt.% epianastrephin (Epi 70). Bars denote mean values (+SE). N (number of cages evaluated with 100 flies each), Median (M), Q1–Q3, and statistical values (W and *p*) are also included on the figure sides. Asterisks denote significant differences (Wilcoxon test).

Traps with a lek caught significantly more immature females (W = 2.40, *p* = 0.0082) than control traps without a stimulus (Figure 3). There were no significant differences in the number of immature females caught in traps with torula, epianastrephin 70, epianastrephin 95, or dimethyl than in control traps (Figure 3). For immature males, traps with a lek caught significantly more flies than control traps without a stimulus (W = 1.84, *p* = 0.0456). There were no significant differences in the number of males caught in traps with torula, epianastrephin 70, epianastrephin 95, or dimethyl than in control traps (Figure 3). When proportions of *A. fraterculus* caught by traps with stimulus were compared (Figure S1), no significant differences were found among them (F3, 66 = 0.28; *p* = 0.8382) or among sexes (F1, 66 = 0.03; *p* = 0.8684), with no significant interaction between these factors (F3, 66 = 0.11; *p* = 0.9563).

#### 3.2.2. Virgin Females

Traps baited with all semiochemicals and the leks caught significantly more virgin females than control traps without a stimulus (Figure 3). Proportions of *A. fraterculus* caught by traps (Figure S1) did not differ among semiochemicals (F_3, 61_ = 0.19; *p* = 0.9052).

#### 3.2.3. Mated Females and Males

Mated females were significantly more attracted to the lek than to the control trap (Figure 3). There were no significant differences in the number of females caught in the traps with epianastrephin 70, epianastrephin 95, and dimethyl compared to the number of flies caught in the control traps (Figure 3). Mated males were significantly more attracted to the trap containing dimethyl, epianastrephin 70, or a lek than to the control trap. Males were equally attracted to the traps containing epianastrephin 95 and the control trap (Figure 3). When proportions of *A. fraterculus* caught by traps with semiochemicals were compared (Figure S1), no significant differences were found among them (F_3, 120_ = 0.26; *p* = 0.8521) or sexes (F_1, 120_ = 0.03; *p* = 0.8685), with no significant interaction between both factors (F_3, 120_ = 0.10; *p* = 0.9600).

## 4. Discussion

Genus or species-specific attractants are one of the best allies in insect-integrated pest management, particularly if they involve the most damaging sex. They are relevant both for pest monitoring and for pest control measures, like those based on attractancy such as baited sprays and attract-and-kill techniques. At present, the monitoring of *A. fraterculus* relies on food-based attractants with poor selectivity, which is a costly tool from an operational perspective. In the search for a more specific attractant, we determined the attractiveness of *A. fraterculus* males and females of different physiological conditions to slow-release polymeric matrix devices with different proportions of three γ-lactone semiochemicals (anastrephin, epianastrephin, and the synthetic γ-lactone analog dimethyl) through electroantennography evaluation and behavioral tests carried out under field cage conditions.

Epianastrephin and dimethyl were EAG active for all the fly conditions considered in the present study. However, the response was higher to epianastrephin than to dimethyl, which might indicate a higher affinity of the binding site of the molecule in the antennal chemoreceptor. Moreover, the response of the flies exposed to both compounds was similar for males and females. This similarity was expected, taking into account the lek mating system of *A. fraterculus* in which males congregate to attract females [22,29]. Thus, any aggregation signal, in a sexual context, should attract both sexes. The response of the antenna of *A. fraterculus* to male-borne volatiles was already evaluated in 10/20 day-old virgin females [18]. Here, we extended the study to males and females of different physiological conditions and focused on synthetic epianastrephin and its analog (dimethyl) to find, in both cases, a positive response.

The strength of the *A. fraterculus* antenna’s responsiveness was conditioned by its reproductive status, being greater for immature flies. Although it was generally assumed that the antenna should always respond in a similar way to a given stimulus, some recent studies show that the antennal response varies throughout the life of the insects [40]. Three receptors involved in the perception and recognition of odorant stimuli [the odorant receptors (ORs), the ionotropic receptors (IRs), and gustatory receptors (GRs)] have been described for the insect chemosensory system. These receptors allow the detection of a wide range of stimuli, including pheromones [41,42]. The response of insects to various external stimuli is regulated by changes in gene expression of these receptors when the insect enters specific physiological conditions [43]. For instance, expression patterns of receptor genes (most IRs and ORs) of the antennae of *B. dorsalis* changed according to a physiological condition, including feeding status, time of day, and mating status [35]. Specifically, gene expression decreased after mating in female flies, showing that olfactory responses to sexual signals are switched off very rapidly after mating. Interestingly, this expression may be correlated with substances present in the male ejaculate, as was found for *A. ludens* [44]. Here, the greater antenna responsiveness of immature *A. fraterculus* could be related to greater gene expression of receptors involved in olfactory response to sexual signals.

The behavioral response of *A. fraterculus* to the semiochemicals differed according to their physiological condition, particularly in the case of females. Response of flies to a particular odor may change throughout the adult stage [14] and is linked to their sexual condition [1]. In general, insects become more attracted to sexual signals (sex pheromone) when they are in search of mating partners, and, as they mature and mate, there is a switch towards oviposition-site cues (fruit odors). In this study, we found that virgin females of *A. fraterculus* showed a significant response to the synthetic semiochemicals in search of males. This response was not significant after mating, probably as a result of responding to fruit odors in search of oviposition sites. This mating-induced change in the olfactory preference of female response from male pheromone to fruit stimuli after mating (“behavioral switch”) was reported for *C. capitata* [45] and *B. tryoni* [46]. More specifically, behavioral studies on the *Anastrepha* species (*A. ludens* and *A. obliqua*) recently demonstrated a clear preference for the male’s sexual pheromone over host volatiles when unmated flies are offered to choose between both stimuli [47]. Although little is known about the underlying mechanism of the behavioral switch, this behavior has been related to the male ejaculate transferred from males to females by the above authors. Our speculation about changes in *A. fraterculus’s* response to the odors throughout its adult stage is supported by behavioral [48] and genetic studies [49]. In the first case, *A. fraterculus* females not only were attracted to fruit volatiles of *Acca sellowiana* and *Psidium cattleianum* when they were mated, but they also showed a clear response to the odor blends of these native host fruits at both electrophysiological and behavioral levels [48]. In the second case, genetic studies reported differential gene expressions of odorant-binding proteins (OBPs) according to the reproductive status of *A. fraterculus* [49]. OBPs have specific affinities to pheromonal and some food odorants and have been proposed as responsible for triggering olfactory responses in insects [50]. According to Campanini et al. [49], expression levels of some OBP genes (*OBP56a*, *OBP99c,* and *OBP83cd*) changed significantly after mating in *A. fraterculus* females, from down-regulated in virgin flies to up-regulated in mated flies. Thus, up-regulated genes in these post-mating females seem to be involved in finding oviposition sites vs. up-regulated genes in virgin females that are probably involved in pheromone perception. Further EAG and behavioral evaluations combining sex pheromones and fruit volatiles can help to confirm our hypothesis.

Interestingly, mated females of *A. fraterculus* were attracted to the leks but not to the semiochemicals. Based on the previous hypothesis, mated females should be seeking fruit to lay their eggs. The chemical characterization of *A. fraterculus’s* male-borne volatiles revealed the presence of volatiles that are also released by host fruits, such as limonene, (E,E)-α-farnesene, and (E,Z)-3,6-nonadien-1-ol [18,32,51]. Therefore, mated females looking for ovipositing sites could be responding to fruit odors present in the male volatiles, not specifically as a result of sexual stimulation but as a response to fruit odors. In this context, similarities between the pheromone composition of *A. fraterculus* males and volatiles of its preferred host fruit, for example, guava [48,52], could be acting as a sensory trap in which males, by releasing fruit odors, attempt to attract mated females and therefore enhance their reproductive success. These multiple stimuli for attraction elicited by the males’ volatiles encourage the design of a blend combining fruit attractants, such as E-β-ocimene, limonene, or other fruit compounds, with epianastrephin to improve its attractiveness, especially to mated females. The host plant component (Z)-non-3-en-1-ol could be another option since it is the unique compound from the male pheromone shared among four *Anastrepha* species [9] and increased female response of *A. ludens* when offered in combination with epianastrephin [53].

The behavioral response of *A. fraterculus* males to the semiochemicals also differed according to their physiological condition. Mature but not immature males were attracted to the semiochemicals (dimethyl and epianastrephin 70). Given the importance of lekking behavior for the reproduction of this fly species, the attractiveness of semiochemicals to mature males is probably a strategy to find other males to congregate in leks. However, mated males were not attracted to epianastrephin 95, a finding that deserves further evaluation.

Response of *A. fraterculus* to the semiochemicals differed according to the test (antennal response by EAG vs. behavioral response in field cage experiments). Epianastrephin elicited stronger antennal response than dimethyl, but both semiochemicals triggered the same behavioral response in the flies. Integration of the information at a different stage in the central nervous system might explain such differences between EAG response and behavior. EAG assays provide the electrophysiological responses of isolated organs and olfactory receptors [9] while behavioral assays (through field cages in our case) allow assessment of the behavioral responses of the flies after integrating all the stimuli and available information [46]. The presence of receptors in other sensory organs could also provide additional information not given by the receptors of the antenna.

From an experimental point of view, this work may have some weaknesses. One is the fact that the flies were released without prior fasting which may have been the reason why torula did not result in higher captures of immature flies in comparison with the food-based attractant control. Second, domestication effects may have potentially influenced our results. Flies used in the tests were from a laboratory colony kept for 25 years, probably explaining the lack of response of mated males to epianastrephin 95. Third, we used McPhail traps that are designed to increase capture through a visual stimulus (yellow base). Future experiments should consider the use of white sticky panels or Steiner-type traps without a visual stimulus. In any case, none of these three weaknesses limits the scope of the study.

In all, this study represents the first approach to evaluate the attractiveness of *A. fraterculus* Brazilian 1 morphotype to epianastrephin, anastrephin, and dimethyl, with promising results. The high attraction found in virgin females clearly confirms the role of epianastrephin as a sexual pheromone. To our knowledge, this is the first report that clearly shows the sexual role of epianastrephin in *A. fraterculus*. Our bioassays showed a promising performance of the analog dimethyl since it elicited the same response in flies as epianastrephin, requires fewer steps to synthesize, and contains one less chiral center than natural pheromones. Dimethyl could be a good early-detection attractant, particularly in pest-free areas such as entry ports in importing countries. Variations of fly attraction according to their physiological status and age is also important for pest management since it contributes to improving available attractants and effective trapping of *A. fraterculus*. The next steps include evaluating the persistence of the attractants under field conditions. (Our preliminary results show that they can elicit attraction for at least 15 days.) Evaluating different concentrations of the stimuli will also be necessary. Here we evaluated plugs with 100 mg of active ingredient (epianastrephin-anastrephin or dimethyl), a lower amount than that used in trimedlure plugs to attract *C. capitata* males (1 gr–3 gr). Further open field studies in commercial orchards in areas with a different abundance of wild populations and in different seasons or regions will provide the ultimate evaluation of the possibility of implementing dimethyl as a new attractant for *A. fraterculus* or other *Anastrepha* species. The addition of fruit compounds may result in greater attraction and deserves further evaluation.

## Figures and Tables

**Table 1:** Results of the general lineal model built to analyze the antenna response (amplified signals expressed as mV) of *A. fraterculus* to the semiochemicals according to their sex (male or female) and physiological condition (age and mated status).

Source of Variation	df	F-Value	*p*-Value
(Intercept)	1	459.12	<0.0001
Sex	1	3.44	0.0659
Physiological condition	2	6.97	0.0013
Semiochemical	1	11.34	0.0010
Sex x Physiological condition	2	3.66	0.0285
Physiological condition x semiochemical	2	0.10	0.9017
Sex x Semiochemical	1	0.08	0.7737

## Data Availability

The data presented in this study are available in article.

## Supplementary Materials

The following supporting information can be downloaded at: https://www.mdpi.com/article/10.3390/insects14020206/s1, Figure S1: Proportion of *Anastrepha fraterculus* caught by traps with semiochemicals. Flies from different physiological conditions (Immature females and males, mature-virgin females, and mature-mated females and males) were separately tested in independent experiments.
