# Supplementary figures and images for: Behavioural and Electrophysiological Response of Anastrepha fraterculus (Diptera: Tephritidae) to a γ-Lactone Synthetic Semiochemical
Source: Insects. 2023 Feb 18;14(2):206. doi: 10.3390/insects14020206 (PMC9958615; doi:10.3390/insects14020206)

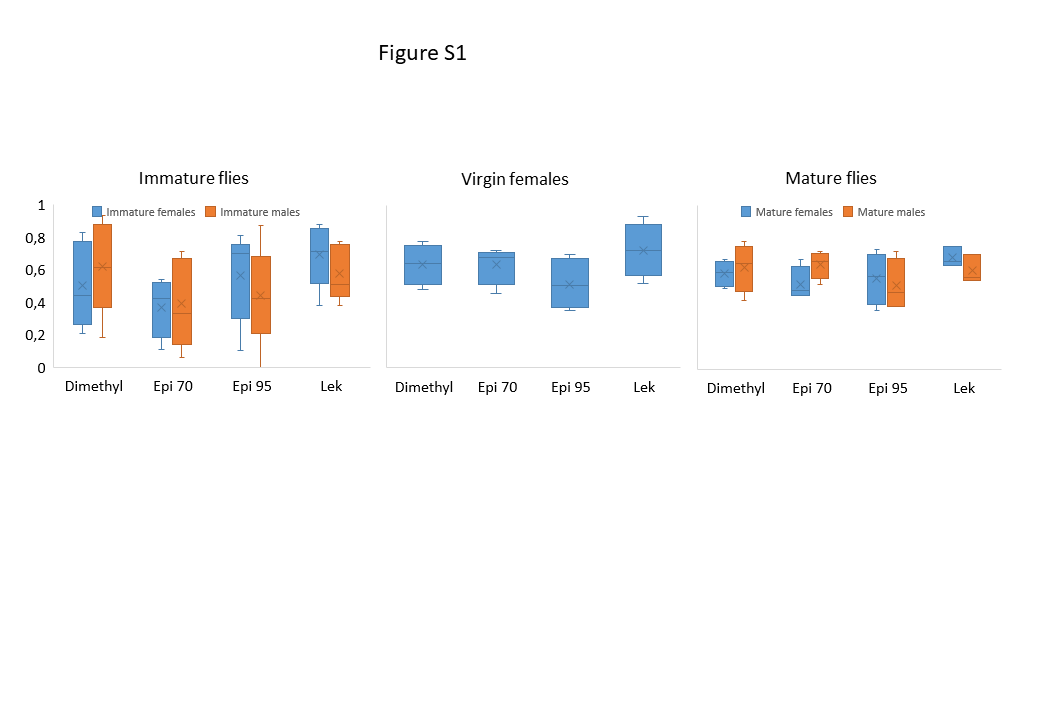

Supplement: Supplementary file 1 [file insects-14-00206-s001.zip › insects-2199776-supplementary figure S1.tif]
